# 2433: Real-time health activity reporting of citizens in Lagos, Nigeria using mHealth app node

**DOI:** 10.1017/cts.2017.150

**Published:** 2018-05-10

**Authors:** Solomon Abiola, Olaoluwa Akinwale, Earl Dorsey, Henry Kautz

**Affiliations:** University of Rochester Medical Center, Rochester, NY, USA

## Abstract

OBJECTIVES/SPECIFIC AIMS: This study sought to develop a mHealth application which was capable of predicting the spread of infectious diseases during the height of the Ebola outbreak in Lagos, Nigeria. Following the success of this primary task, the research then sought to understand behavioral health issues which are indicative of chronic diseases, such as sedentary behaviors and where they occur at a geospatial level in real-time. The results of this study are now being used to develop a larger scale 500 person study in Rochester, NY, USA. METHODS/STUDY POPULATION: During a 3-month period individuals were asked to install a mobile health application known as Node onto the their android device. Consent was done remotely, individuals were recruited through the Lagos University Teaching Hospital, Nigeria Institute of Medical Research, and the University of Lagos. Participants were paid 50 USD/month for each month of study completion, while continuous location data was collected in addition to survey information about participants. RESULTS/ANTICIPATED RESULTS: During the study period 70 individuals enrolled, using this data we were able to create network based models which indicated that diseases were more likely to spread at the beginning of the week, and also indicated who would be most susceptible to being patient zero. In phase 2 we have started to look at behavioral patterns to determine the risk of chronic disease among our study population, by examining their human mobility patterns, since we can determine average sleep patterns, activity patterns using machine learning classifiers, and time spent in traffic—all of which we can visualize in a real-time geospatial manner with higher objectivity than traditional mechanisms for data collection. DISCUSSION/SIGNIFICANCE OF IMPACT: In developing countries, using Nigeria as our example most chronic disease and household studies only enroll a few thousand participants for a country numbering 150 million plus. Using our rapidly available application we were able within 1 week to enroll 70 participants on 1 year of funding, this creates a framework for larger scale public health studies which can be done in developing countries and also demonstrates the value in mHealth which can both answer questions of infectious disease and chronic diseases at the same time. Our results indicate that at an infectious disease level in city environments diseases may be prevented by targeting events early in the week. While at a chronic disease level the lack of reliable power results in less sedentary behavior as individuals seek locations to charge phones, while those with more stable western-like lifestyles have started to exhibit the conditions which cause such outcomes as obesity, which has begun to rise in developing countries. Ultimately, these results serve as a staging point to launch a more wide scale study both in the United States and Nigeria within the year, now that feasibility has been established.

